# Phytofabrication of Selenium Nanoparticles From *Emblica officinalis* Fruit Extract and Exploring Its Biopotential Applications: Antioxidant, Antimicrobial, and Biocompatibility

**DOI:** 10.3389/fmicb.2019.00931

**Published:** 2019-04-30

**Authors:** Lokanadhan Gunti, Regina Sharmila Dass, Naveen Kumar Kalagatur

**Affiliations:** ^1^Molecular Fungal Genetics and Mycotoxicology Research Unit, Department of Microbiology, Pondicherry University, Puducherry, India; ^2^Food Microbiology Division, Defence Food Research Laboratory, Mysuru, India; ^3^Immunology and Toxicology Division, DRDO-BU Center for Life Sciences, Coimbatore, India

**Keywords:** phytofabrication, selenium nanoparticles, *Emblica officinalis*, antioxidant activity, antimicrobial activity, biocompatibility

## Abstract

In the present study, phytofabricated selenium nanoparticles (PF-SeNPs) were prepared from aqueous fruit extract of *Emblica officinalis* in a facile, green, economic, tactic and eco-friendly way. The aqueous fruit extract of *E. officinalis* was found to be rich with various secondary metabolites including phenolics (59.18 ± 2.91 mg gallic acid equivalents/g), flavonoids (38.50 ± 2.84 mg catechin equivalents/g), and tannins (44.28 ± 3.09 mg tannic acid equivalents/g) and determined that highly appropriate for the biosynthesis of nanoparticles. The facile phytofabrication of PF-SeNPs was confirmed by UV-visible and FTIR spectroscopic analysis. The XRD pattern and Raman spectroscopy showed that synthesized PF-SeNPs were amorphous in nature. The Zeta potential analysis confirmed that PF-SeNPs were negatively charged (-24.4 mV). The DLS analysis revealed that PF-SeNPs were in nano size and less aggregated with poly-dispersity index of less than 0.2. The SEM images depicted that PF-SeNPs were spherical in shape. The EDX analysis revealed that PF-SeNPs were constituted with Se (61.60%), C (29.96%), and O (4.41%). The HR-TEM analysis determined that PF-SeNPs were in nano size with an average diameter of 15–40 nm. The PF-SeNPs have offered fascinating bio-potential applications, such as antioxidant, antimicrobial and biocompatibility. They have also exhibited dose-dependent free radical scavenging activity, and EC50 was determined as 15.67 ± 1.41 and 18.84 ± 1.02 μg/mL for DPPH and ABTS assays, respectively. The PF-SeNPs has also shown the wide range of antimicrobial activity on foodborne pathogens, and it was found to be highly efficient on fungi followed by Gram-positive and Gram-negative bacteria. The biocompatibility of PF-SeNPs was assessed in N2a cells with much higher IC50 value (dose required to inhibit 50% of cell viability) compared to sodium selenite. Also, mitochondrial membrane potential (MMP) and caspase-3 were much less altered on treatment of PF-SeNPs related to sodium selenite. The cytotoxic studies clearly determined that PF-SeNPs was much less toxic and safer related to sodium selenite. Thus, PF-SeNPs could find suitable application as antioxidant and antimicrobial agent in food, biomedical, and pharmaceutical industry.

## Introduction

Antibiotic resistance and food safety have become two of the major health apprehensions for the public, government, and regulatory agencies in the last two decades (Sundararaj et al., 2019). The infectious diseases are the primary causes of deaths that occur worldwide. Ever since the advent of antibiotics, morbidity, and mortality rate of infectious diseases are being mitigated. However, nowadays, there is an upsurge in antibiotic-resistant microorganisms, which is an emerging utmost concern. Also, Food and Agriculture Organization (FAO) of the United Nations estimate a loss of 25% of the agronomic products worldwide owed to fungal infestation and is measured as major threat to food industry (Bryden, 2007; Kalagatur et al., 2018c,d). Particularly, fungal secondary metabolites (mycotoxins) are highly poisonous and could cause a variety of ailments in humans and farm animals (Mudili et al., 2014). Further, the use of synthetic food additives is one of the foremost concerns in food industries because of their ability to incite digestive disorders and carcinogenicity (El-Wahab and Moram, 2013). Thus, there is a necessity to design substitutive compounds with antioxidant and antimicrobial properties that do not induce antimicrobial resistance and detrimental effects on human and animal health and can be used by the food industries in compliance to food safety.

The recent boom in nanotechnology has fortunately provided us an indefinite range of applications in biomedicine and food science. Particularly, nanoparticles have the extensive range of applications in biological sciences, i.e., pharmaceuticals, medical diagnosis, cosmetics, agriculture and food industry, etc. (Kalagatur et al., 2018a; Siddaiah et al., 2018). In the last decade, several pharmaceutical companies have obtained the consent of nano-formulation based drugs and diagnostics from the Food and Drug Administration (FDA) and World Health Organization (WHO) (Mittal et al., 2014). Furthermore, nanomaterials have also found an appropriate role in the agro-food-feed sectors; for example, crop production, nutritional properties, enhancement of water quality, food packaging, etc.

The most routinely synthesized nano-antimicrobials are made up of silver, gold, titanium, zinc, cadmium, gadolinium, selenium, copper, etc. However, due to the high cost of silver, gold and copper metals, their use in biomedical and food sciences is limited. Also, cadmium, titanium, gadolinium, and zinc are unacceptable due to their high toxic nature (Zuverza-Mena et al., 2017; Sohal et al., 2018). Among the nanomaterials, selenium nanoparticles (SeNPs) have drawn attention and are widely accepted in biomedicine and food science due to their low toxicity and high biocompatibility (Tran et al., 2015). Captivatingly, selenium is a key factor in the formation of selenoproteins, which are vital antioxidants like thioredoxin reductase, glutathione peroxidase, and deiodinase (Rotruck et al., 1973). Selenium is one of the trace minerals, which is essential for the maintenance of human health, with approximately 40–300 mg as daily nutritional supplement for an adult (Rayman, 2005). Further, SeNPs have potent free radicals scavenging effects, both in *in vitro* as well as *in vivo* conditions and protect DNA from oxidative damage (Battin et al., 2011). Along with the aforementioned properties, several studies have reported that the SeNPs possess anti-carcinogenic activity against several types of cancers (Huang et al., 2013). Also, SeNPs show unique antimicrobial activities against *Candida albicans* (Kheradmand et al., 2014), *Proteus mirabilis* and *Pseudomonas aeruginosa* (Shakibaie et al., 2015). Accordingly, SeNPs are highly acceptable and recommended for use in biomedical and food science.

Selenium nanoparticles can be synthesized by physical, chemical, and biological approaches. The chemical and physical methods need high thermal conditions, hazardous chemicals and acidic pH, which is extremely toxic and unsafe for biological applications (Iranifam et al., 2013). Whereas, biological synthesis of SeNPs is safe, eco-friendly, inexpensive and non-toxic (Wadhwani et al., 2016). Moreover, biologically made SeNPs are more stable due to the natural coating of organic materials over the surface, which do not allow nanoparticles to be aggregated with the time period (Park et al., 2011). The use of plant extracts for the synthesis of nanoparticles might be beneficial over microbial synthesis by eliminating the extravagant procedures for maintaining cultures. Despite the fact, that a large number of plants are reported for nanoparticle synthesis, only few reports are available on phytogenic synthesis of SeNPs. These studies include synthesis using leaf extract of *Capsicum annuum* (Li et al., 2007), seed extract of fenugreek (Ramamurthy et al., 2013), leaf extract of lemon (Prasad et al., 2013), dried fruit extract of *Vitis vinifera* (Sharma et al., 2014), leaf extract of *Terminalia arjuna* (Prasad and Selvaraj, 2014), flower extracts of *Bougainvillea spectabilis* (Deepa and Ganesan, 2014), leaf extract of *Leucas lavandulifolia* (Kirupagaran et al., 2016), leaf extract of *Clausena dentate* (Sowndarya et al., 2017), aqueous extract of *Allium sativum* (Anu et al., 2017), leaf extracts of *Diospyros montana* (Kokila et al., 2017), leaf extracts of *Psidium guajava* (Alam et al., 2018), polysaccharides from *Lycium barbarum* and green tea extracts (Zhang et al., 2018). In this study, we report for the first time, a quick synthesis of phytofabricated selenium nanoparticles (PF-SeNPs) from fruit extract of *Emblica officinalis* and establishment of biocompatibility of PF-SeNPs relating with cytotoxicity of precursor sodium selenite.

*Emblica officinalis* is also known as *Phyllanthus emblica*, which belongs to the family Phyllanthaceae and genus *Phyllanthus*, whose edible fruits are widely used in Indian Ayurvedic medicine (Dharmananda, 2003). There are abundant benefits of *E. officinalis*, which include, antibacterial (Philip et al., 2012), antifungal (Mehmood et al., 1999), antioxidant and cardioprotective activities (Golechha et al., 2012). The *E. officinalis* fruits are a rich source of hydrolyzable phenolics, flavonoids, and tannins that play a key role in reducing and regulating the shape of nanoparticles (Ankamwar et al., 2005).

In the present study, phytofabricated-SeNPs (PF-SeNPs) were synthesized using aqueous fruit extracts of *E. officinalis* by green, low-cost and simple reduction method. The as-synthesized PF-SeNPs were preliminarily characterized and confirmed by UV–visible spectroscopy. The particle sizes of PF-SeNPs were determined by dynamic light scattering (DLS) analysis. The nature of PF-SeNPs was confirmed by Fourier transform infrared spectroscopy (FTIR), Raman spectroscopy, and X-ray diffraction (XRD) analysis. The shape, size, and chemical composition of PF-SeNPs were determined by scanning electron microscopy (SEM), high-resolution transmission electron microscopy (HR-TEM), and energy dispersive X-ray analysis (EDX) analysis, respectively. In conclusion, potential biological applications of PF-SeNPs were explored by antibacterial, antifungal, antioxidant and biocompatibility assays.

## Materials and Methods

### Chemicals and Reagents

Sodium selenite (98%), 2,2-diphenyl-1-picrylhydrazyl (DPPH), 3-(4,5-dimethylthiazol-2-yl)-2,5-diphenyltetrazolium bromide) (MTT), Tween 80, methanol, acetate buffer, tetracycline, nystatin, rhodamine 123, caspase-3 kit, Dulbecco’s modified Eagle media (DMEM), Folin-Ciocalteu reagent, 2,2-azinobis-(3-ethylbenzothiazoline-6-sulfonate) (ABTS), Dulbecco’s phosphate buffered saline pH 7.4 (DPBS) and fetal bovine serum (FBS) were obtained from Sigma-Aldrich (Bengaluru, India). The microbial culture media, including Muller Hinton Broth (MHB), Muller Hinton Agar (MHA), brain heart infusion (BHI) broth, sabouraud dextrose agar (SDA), and sabouraud dextrose broth (SDB) were obtained from HiMedia (Mumbai, India). The live/dead dual staining assay kit was purchased from Thermo Fisher Scientific (Bengaluru, India). The plastic and glassware were obtained from Nunc and Borosil, respectively (Bengaluru, India). The other chemicals used in the study were belonged to analytical grade and were obtained from Merck Millipore (Bengaluru, India).

### Collection and Preparation of *E. officinalis* Fruit Aqueous Extract

The fresh fruits of *E. officinalis* were collected from the local agriculture market, Pondicherry, India. They were washed thoroughly with distilled water, deseeded using a sterile knife and the edible part of the fruit was used to prepare fruit extract. Briefly, 100 g of edible fruit was ground well in a mortar using water (w/v, 1:2). The solution obtained was filtered through Whatman No. 1 filter paper, stored at 4°C and further used for chemical profile analysis and nanoparticle preparation.

### Chemical Profile of Aqueous Fruit Extract of *E. officinalis*

Plants and its sources contain a wide range of secondary metabolites, and these were considered as potential reducing substances for biogenic production of nanoparticles (Akhtar et al., 2013). The total phenolic, flavonoid and tannin contents of *E. officinalis* fruit extract were determined in order to evaluate its suitability in biogenic production of nanoparticles.

#### Estimation of Total Phenolics

The total phenolic content of *E. officinalis* fruit extract was determined by Folin-Ciocalteu assay (Kalagatur et al., 2018b). Gallic acid was considered as a reference phenolic compound and the obtained result was stated as mg of gallic acid equivalents per g of *E. officinalis* fruit extract (mg GAE/g). Briefly, 0.5 mL of fruit extract was diluted thrice with distilled water and blended with 0.5 mL of 7.5% sodium carbonate solution and 0.25 mL of Folin-Ciocalteu reagent. The obtained blend was incubated for 30 min at 27 ± 2°C under dark and absorbance was recorded at 765 nm using a plate reader (Synergy H1, BioTek, United States).

#### Estimation of Total Flavonoids

The total flavonoid content of *E. officinalis* fruit extract was determined by aluminum chloride colorimetric assay (Kalagatur et al., 2018b). Catechin was considered as a reference flavonoid compound and the obtained result was stated as mg of catechin equivalents per g of *E. officinalis* fruit extract (mg CE/g). Briefly, 0.5 mL of fruit extract was blended with 70 μL of sodium nitrite solution (5%) and incubated at 27 ± 2°C for 5 min. Later, the mixture was blended with 0.5 mL of sodium hydroxide (1 M), 0.15 mL of aluminum chloride (10%) and 1.3 mL of deionized water. The reaction mixture was incubated for 5 min at 27 ± 2°C and absorbance was measured at 415 nm using a plate reader (Synergy H1, BioTek, United States).

#### Estimation of Total Tannins

Total tannin content was estimated spectrophotometrically as per procedure of Price and Butler (1977). Tannic acid was considered as reference tannin compound and the obtained result was stated as mg of tannic acid equivalents per g of *E. officinalis* fruit extract (mg TAE/g). Briefly, 4 mL of fruit extract was blended with 3 mL of 0.1 M FeCl_3_ in 0.1 N HCl and 3 mL of 0.008 M K_3_Fe(CN) and incubated in the dark for 15 min at 27 ± 2°C. Later, the optical density was measured at 720 nm using a multimode reader (Synergy H1, BioTek, United States).

### Synthesis of Phytofabricated Selenium Nanoparticles (PF-SeNPs)

Briefly, 2 mL of aqueous fruit extract of *E. officinalis* was added dropwise into 10 mL of 10 mM sodium selenite under magnetic stirring condition. Next, reaction mixture was allowed for reduction in dark condition at 27 ± 2°C and 120 rpm on orbital shaker for 24 h and observed for color change.

### Characterization of Biogenic PF-SeNPs

#### UV–Visible Spectroscopy

The bio-reduction of sodium selenite by aqueous fruit extract of *E. officinalis* was monitored by observing the color change. After the formation of brick-red color, absorbance of the nanoparticles was measured at wavelength ranging between 200 and 800 nm with 1 nm wavelength intervals using UV–visible spectrophotometer (Agilent-Cary 60, United States).

#### Fourier Transform Infrared (FTIR) Spectroscopic Analysis

Fourier Transform Infrared spectrum was used in order to confirm the presence of various reducing and stabilizing functional groups of metabolites in aqueous fruit extract of *E. officinalis* and to detect their possible role in fabrication of PF-SeNPs. Briefly, PF-SeNPs solution was dried out and ground into a homogeneous powder, and spectra were attained at 400–4,000 cm^-1^ wavenumbers against potassium bromide background using the spectrophotometer (Thermo Nicolet 6700, United States).

#### X-Ray Diffraction Analysis

The phase structure of PF-SeNPs was observed by XRD analysis using X’Pert PRO diffractometer in Debye-Scherrer configuration (PANalytical, Spectris Technologies Pvt. Ltd., India) attached with Cu anode as a source of X-rays and Ni filter was used to attenuate pipeline of Cu Kβ. The Cu Kα radiation was generated at a wavelength of 1.5406 Å by operating the instrument at 40 kV and 40 mA. The measurement was recorded by using a 0.5 mm glass capillary over a 2θ range of 10–80° with a scanning rate of 0.05° at a time interval of 1 s. The Scherrer equation was applied to calculate the average dimension (*L*_a_) of crystalline domains,

$$L_{a}\;=\;\frac{K\text{λ}}{\text{β}\;\cos({\text{θ}}_{Bragg})}$$

Where, *L*_a_ was the average dimension of crystalline domains, *K* was the non-dimensional shape factor and usually considered as 0.9, β was the full width at half maximum (FWHM) in 2θ value, and λ was the wavelength at which Cu *K*α radiation was generated.

#### Raman Spectroscopic Analysis

Raman spectroscopy was used to explore the nature of nanomaterials, which is recognized as a perfect technique that even detects slight variations. Briefly, dried PF-SeNPs were placed as a thin film on aluminum foil and spectra were recorded at 785 nm excitation and spectral range of 150–1,200 cm^-1^ with an interval of 10 s using Renishaw’s in Via Raman microscope (Renishaw, India).

#### Dynamic Light Scattering and Zeta-Potential Analysis

This technique was used to determine the hydrodynamic size of particles by focusing a monochromatic light on to the solution, which causes a Doppler shift. Once the laser light hits the moving particles, it changes the wavelength of the incoming light and scatters the light at an angle. This angle of scattered light is inversely propositional to the size of the particle. The size distribution of PF-SeNPs was monitored at 27 ± 2°C using Zetasizer (Nano ZS, Malvern Instrument, United Kingdom). The Zeta-potential of the nanoparticles was measured to find the surface charge on it.

#### Scanning Electron Microscopic and Energy-Dispersive X-Ray Analysis

Scanning electron microscopy was carried to know the shape, surface morphology and size of the nanoparticles. Briefly, PF-SeNPs suspension was air dried before loading them to sample holders. Later, PF-SeNPs were coated with gold using sputter coater in a vacuum and further, images were taken at 20 kV and different magnifications using SEM (Icon Analytical, Quanta 250, FEI, United States). Energy-dispersive X-ray (EDX) analysis was performed at 20 kV in order to know the elemental compositions of nanoparticles. The PF-SeNPs were dissolved in absolute ethanol and one drop of the suspension was placed on a sample loading grid, evenly dried and element analysis was performed.

#### High-Resolution Transmission Electron Microscopy Analysis

The shape and size of PF-SeNPs was determined by HR-TEM analysis. Briefly, one mg of PF-SeNPs was suspended in 1 mL of ethanol and subjected to sonication for 15 min. Subsequently, a drop of supernatant dispersion was collected and placed on the copper grid, and images were captured at different magnifications using HR-TEM (JEM-2100, JEOL Ltd., Tokyo, Japan).

### Biopotential Applications of PF-SeNPs

#### Antioxidant Assay

Free radical scavenging activity of PF-SeNPs was determined by DPPH and ABTS radical scavenging assays as per earlier reported methods (Kumar et al., 2016; Sellamani et al., 2016).

In DPPH radical scavenging assay, various concentrations of PF-SeNPs (up to 100 μg) was blended with 0.5 mL of DPPH solution (250 μM in methanol) and 1 mL of 0.1 M acetate buffer and final volume was made up to 3 mL with methanol. The reaction mixture was shaken thoroughly and left in the dark for 30 min at 27 ± 2°C. Then the absorbance was measured at 517 nm using UV-Visible spectrophotometer (Agilent-Cary 60, United States). The reaction mixture without PF-SeNPs was used as control and ascorbic acid was used as standard.

In ABTS radical scavenging assay, ABTS radical solution was produced by reaction between 7 mM of ABTS and 2.45 mM of potassium persulfate (1:1) in water and incubated at 27 ± 2°C under the dark for 12 h. Further, the optical density of ABTS radical solution was adjusted to 0.7 at 734 nm by diluting with methanol. Subsequently, various concentration of PF-SeNPs (up to 100 μg) was added to 3 mL of ABTS solution (0.7 optical density) and incubated at 27 ± 2°C for 6 min under dark and optical density was measured at 734 nm using UV-visible spectrophotometer (Agilent-Cary 60, United States). The ABTS solution without test sample was used as control and ascorbic acid was used as standard.

The DPPH and ABTS radical scavenging activity of the test sample was calculated using the formula,

$$\mathrm{DPPH}\;(\mathrm{or})\;\mathrm{ABTS}\;\mathrm{radical}\;\mathrm{scavenging}\;\mathrm{activity}\;(\%)\;=\;\frac{\left(1\;-\;A_{TS}\right)}{A_{C}}\;\times \;100$$

Where, *A*_TS_ and *A*_C_ were absorbance of the test sample and control, respectively.

#### Antimicrobial Activity

##### Microbial culture maintenance

Antimicrobial activity of PF-SeNPs was carried out on foodborne pathogens, including bacteria (*Escherichia coli* MTCC 41*, Listeria monocytogenes* MTCC 657, *Staphylococcus aureus* MTCC 96, and *Enterococcus faecalis* MTCC 439) and fungi (*Aspergillus brasiliensis* MTCC 1344*, A. flavus* MTCC 1883, *A. oryzae* MTCC 634, *A. ochraceus* MTCC 10276, *Fusarium anthophilum* MTCC 10129, and *Rhizopus stolonifer* MTCC 4886). Bacteria were grown in BHI broth for 24 h at 37°C and optical density was determined at 600 nm using the UV-visible spectrophotometer (Agilent-Cary 60, United States). The optical density (OD) of the broth was adjusted to 0.5 McFarland standard with sterile phosphate-buffered saline, pH 7.4 (PBS) and used in further studies. Similarly, the fungal isolates were grown in SDA Petri plates for 7 days at 28°C and fungal spores were collected in sterile peptone broth containing 0.001% Tween 80 by using a soft scraper. The spore count was determined by hemocytometer, and its count was adjusted to 1 × 10^6^ per mL and used in further studies.

##### Micro-well dilution method

Antimicrobial activity of PF-SeNPs on foodborne pathogens was determined by micro-well dilution technique as per instructions of Clinical and Laboratory Standards Institute (CLSI) (Kalagatur et al., 2015).

For antibacterial activity, different concentrations of PF-SeNPs and 10 μL of active bacterial culture of 0.5 McFarland standards were added to the wells of 96-well plate, and final volume was adjusted to 100 μL with MHB. The plates were incubated at 37°C for 24 h and OD at 600 nm was determined using the spectrophotometer. The concentration of PF-SeNPs at which no increase in OD was noticed as minimum inhibitory concentration (MIC). Further, 10 μL of sample was collected from the wells and spread plated on MHA Petri plates and incubated for 24 h at 37°C. The concentration of PF-SeNPs at which complete bacterial growth was not observed, was defined as minimum bactericidal concentration (MBC). Tetracycline was used as a standard antibacterial agent.

For antifungal activity, different concentrations of PF-SeNPs and 10 μL of fungal spores (1 × 10^6^ per mL) were added to wells of 96-well plate, and final volume was adjusted to 100 μL with SDB and incubated at 28°C for 3 days. The concentration of PF-SeNPs, at which no visible fungal growth was observed, served as MIC. Further, 10 μL of sample was collected from the wells and inoculated on SDA Petri plates and incubated for 3 days at 28°C. The concentration of PF-SeNPs at which no fungal growth was observed, served as minimum fungicidal concentration (MFC). Nystatin was used as a standard antifungal agent.

#### Biocompatibility Assay

Finally, biocompatibility of PF-SeNPs was assessed by relating with the precursor (sodium selenite) in *in vitro* cell line model by various cytotoxicity assays, including MTT, live/dead dual staining, mitochondrial membrane potential (MMP), and caspase-3 assays. The toxic effect of sodium selenite and PF-SeNPs on cell viability was studied by MTT and live/dead dual staining assays (Kalagatur et al., 2017, 2018b). The MMP was crucial for ATP synthesis and maintaining the other functionalities of cells. The effect of sodium selenite and PF-SeNPs on MMP of cells was studied by rhodamine 123 staining assay (Kalagatur et al., 2017). Caspase-3 is caspase protein, and its activation by both extrinsic (death ligand) and intrinsic (mitochondrial) pathways leads to apoptotic cell death. The caspase-3 activity was determined using caspase-3 assay kit (Kalagatur et al., 2018b).

##### Cell culture and treatments

The cell line, N2a (*Mus musculus* neuroblastoma) was obtained from National Centre for Cell Sciences (NCCS), Pune, India. The cells were sub-cultured in DMEM supplemented with 10% FBS, 50 μg/mL of streptomycin and 50 mU/mL of penicillin in humidified incubator at 5% CO_2_ and 37°C. The cells were grown in 75 cm^2^ cell culture flask, and confluent cells were used in further experiments. The stock solution of sodium selenite and PF-SeNPs were prepared in DMSO (0.5%). Further, working concentration of sodium selenite and PF-SeNPs were made in DMEM devoid of FBS and concentration of DMSO was maintained at <0.01% throughout the study.

Approximately, 1.5 × 10^4^ confluent cells were seeded in 96-well cell culture plates and allowed to adhere for overnight. Then, the cells were treated distinctly with different concentrations of sodium selenite and PF-SeNPs in DMEM medium devoid of FBS for 24 h. The cells treated alone with DMEM devoid of FBS was considered as control. The cells treated with H_2_O_2_ was considered as a positive control. Subsequently, plates were distinctly used for MTT, live/dead, MMP, and caspase-3 assays.

##### MTT assay

Succeeding, treatments and incubation were as detailed in section “Cell Culture and Treatments.” The media was replaced with 10 μL of MTT dye (5 mg/mL) and 90 μL of DMEM devoid of FBS and incubated for 4 h at 37°C. Finally, the medium containing MTT was aspirated and substituted with DMSO for 30 min to solubilize the formazan crystals and absorbance was measured at 570 nm using multiplate reader (Synergy H1, BioTek, United States). The cell viability was expressed with respect to control (100%).

##### Live/dead cell assay

Succeeding, treatments and incubation were as detailed in section “Cell Culture and Treatments.” The cells were washed for twice with DPBS and stained with dual staining dyes (4 μM of ethidium homodimer-1 and 2 μM of calcein AM) and cell viability were determined as per methodology of Kalagatur et al. (2017). Also, images were captured using an inverted fluorescence microscope (EVOS, Thermo Fisher Scientific, United States).

##### MMP assay

Succeeding, treatments and incubation were as detailed in section “Cell Culture and Treatments.” The cells were washed for twice with DPBS and stained with rhodamine 123 (5 μM) in DPBS for 15 min. The fluorescent images were captured under GFP filter of an inverted fluorescence microscope (EVOS, Thermo Fisher Scientific, United States) and optical density was recorded at excitation of 511 nm and emission of 534 nm using multiplate reader (Synergy H1, BioTek, United States). The results were expressed with respect to control (100%).

##### Caspase-3 assay

Succeeding, treatments and incubation were as detailed in section “Cell Culture and Treatments.” The cells were washed twice with DPBS and treated with caspase-3 kit as per manufacturer’s instructions and absorbance was measured at an excitation of 360 nm and emission of 460 nm using a plate reader (Synergy H1, BioTek, United States). The results were expressed with respect to control (100%).

### Statistical Analysis

All the experiments were done independently in triplicates, and results were expressed as mean ± SD. The data were processed by one-way ANOVA and the statistical difference were estimated by Tukey’s multiple comparison test using software GraphPad Prism trial version 7 (San Diego, United States). The *p*-value was considered as a significant at ≤0.05.

## Results and Discussion

### Chemical Profile of *E. officinalis* Fruit Extract

**Figure 1:**
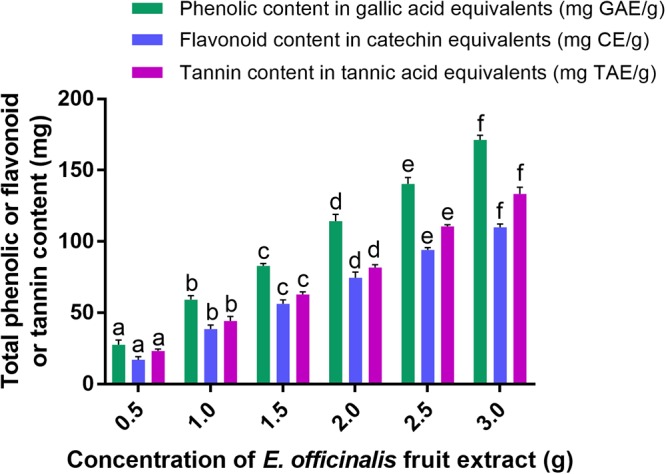
Total phenolic, flavonoid, and tannin contents in aqueous fruit extract of *E. officinalis*. The experiments were executed independently in triplicates and data was expressed as mean ± SD. The statistical difference between the test samples were assessed by Tukey’s multiple comparison test and *p* ≤ 0.05 was considered as a significant. The bar graphs with different alphabetic letters within the respective study were significant.

Generally, plant sources are eco-friendly, non-toxic, and highly esteemed based on their utility in bio-medicine and food sciences. Especially, plant secondary metabolites are highly appreciated and have already been reported in prodigious claims, i.e., antioxidant, antimicrobial, anticancer properties, etc. (Golechha et al., 2012; Philip et al., 2012; George et al., 2016; Muniyandi et al., 2017). Also, plant secondary metabolites are reported as potential candidates for biogenic production of nanoparticles and the method was highly satisfactory and being termed as green synthesis tactic (Akhtar et al., 2013). In the present study, *in vitro* assays for total phenolic, flavonoid and tannin contents of *E. officinalis* fruit extract are shown in Figure 1. The results revealed that *E. officinalis* fruit extract contain decent amount of phenolics (59.18 ± 2.91 mg GAE/g), flavonoids (38.50 ± 2.84 mg CE/g), and tannins (44.28 ± 3.09 mg TAE/g).

In support of our results, Liu et al. (2008a) have determined 62.5 ± 0.7, 203.5 ± 2.1, 269.3 ± 0.4, and 439.9 ± 1.3 mg GAE/g of total phenolic contents in aqueous, butanol, ethyl acetate, and ethyl ether extracts of amla fruit, respectively. Additionally, Liu et al. (2008b) have assessed the phenolic and flavonoid contents of methanolic extracts of amla fruit from six different regions of China and determined in the range from 81.5 to 120.9 mg GAE/g and 20.3 to 38.7 mg quercetin equivalents (QE)/g, respectively. Similarly, Pientaweeratch et al. (2016) from Thailand have determined total phenolic content of 362.43 ± 11.2 mg GAE/g and total flavonoid content of 21.04 ± 0.67 mg QE/g in ethanolic extract of amla. Further, Nambiar et al. (2015) carried out experiments to compare the phytochemical analysis of methanolic extracts of fruit, pulp and seeds of *E. officinalis*, where they determined the total phenolic content, flavonoid content and tannin content in the range of 6.00 ± 0.01–6.50 ± 0.10 μg GAE/mg of dry weight, 71.28 ± 1.00–72.35 ± 0.04 μg QE/mg of dry weight and 6.06 ± 0.01–111.26 ± 0.01 μg TAE/mg, respectively. However, insufficient reports are available in support of our study, especially on tannin content of *E. officinalis*. Typically, secondary metabolites of plants depend on various factors, i.e., genetics of plant, weather and harvesting conditions, nutrition availability, extraction technique, etc. (Verma et al., 2018). Therefore, obtained the quantity of phenolics, flavonoids and tannins in the present study was quite dissimilar with earlier reports. Overall, the present as well as earlier reports accomplish that *E. officinalis* fruit extract is rich with phenolics, flavonoids, and tannins, and it could be the potential contender for reduction and stabilization of metal ions and green synthesis of nanoparticles (Akhtar et al., 2013).

### Biogenic Synthesis and Characterization of PF-SeNPs

**Figure 2:**
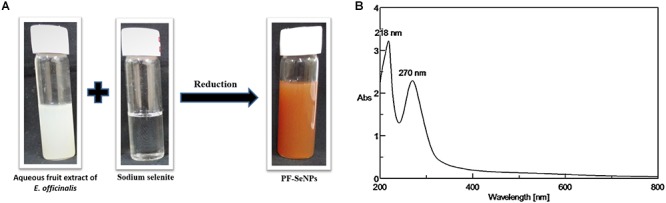
**(A)** Reduction of sodium selenite by aqueous fruit extract of *E. officinalis* to form phytofabricated selenium nanoparticles (PF-SeNPs). **(B)** UV-visible spectra of biogenic PF-SeNPs.

Originally, sodium selenite solution was colorless. The color of sodium selenite turned into brick-red with the addition of *E. officinalis* fruit extract after 24 h (Figure 2A). The formation of brick-red solution was due to the excitation of the surface plasmon resonance and it was an indication for reduction of sodium selenite into elemental selenium. The reduction of sodium selenite into PF-SeNPs can occur by the action of phenolics, flavonoids, and tannins of *E. officinalis* (Ankamwar et al., 2005). The preliminary confirmation of PF-SeNPs formation was concluded by plasmon resonance using UV-Visible spectroscopy. The absorption maximum was observed at 270 nm, which indicate that plant material has reduced and stabilized the PF-SeNPs formation (Figure 2B). The UV-visible absorption maximum in the present study was found to be in agreement with earlier reports. Kokila et al. (2017) have prepared SeNPs from *Diospyros montana* leaf extract by green synthesis approach and observed UV-visible absorption maximum at 261 nm. Similarly, Anu et al. (2017) have green-synthesized SeNPs from *Allium sativum* and noticed UV-visible maximum at 260 nm. Likewise, Fesharaki et al. (2010) have observed UV-visible maximum at 218 and 248 nm (located between 200 and 300 nm) for *Klebsiella pneumoniae* mediated bio-synthesized SeNPs. On the other hand, Shah et al. (2010) have synthesized polyvinyl alcohol-stabilized SeNPs by wet chemical method involving a reaction of acetone and noticed UV-visible maximum at 270 nm. The study concluded that sodium selenite was successfully converted into PF-SeNPs by reduction action of aqueous fruit extract of *E. officinalis*.

**Figure 3:**
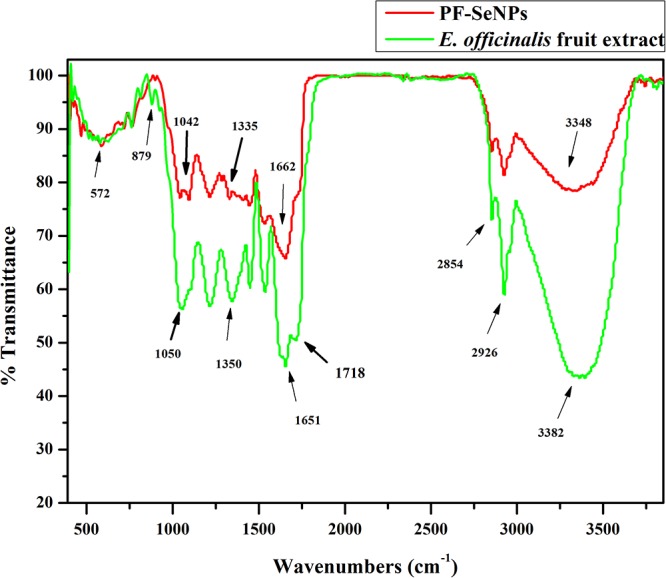
Fourier transform infrared spectroscopy (FTIR) of aqueous fruit extract of *E. officinalis* and phytofabricated selenium nanoparticles (PF-SeNPs).

Further, FTIR spectroscopic analysis was performed to confirm the feasible role of *E. officinalis* fruit extract in the bio-synthesis of PF-SeNPs. FTIR allows the determination of the functional groups that exist on the surface of nanoparticles by measuring the vibrational frequencies of chemical bonds. The obtained molecular data facilitates to establish structural and conformational changes of the co-ordinating self-assembled functional groups on the surface of nanoparticles (Movasaghi et al., 2008). In the present study, FTIR spectra of aqueous fruit extract of *E. officinalis* and PF-SeNPs have been detailed in Figure 3. FTIR spectrum of aqueous fruit extract of *E. officinalis* has presented multiple intense peaks at 3,382, 2,926, 2,854, 1,718, 1,651, 1,448, 1,350, 1,211, 1,050, 879 and 572 cm^-1^, which corresponds to attendance of –OH group, stretching vibration of aliphatic C–H, carboxylic acid O–H, carbonyl C=O stretch, amide I vibrations, C=C aromatic, CH_3_ C–H bending in alkyls, R–O–R (ether), superposition in the plane of C–H bending of polysaccharide, C–C stretching vibration and OH bending of the phenolic groups, respectively (Ankamwar et al., 2005; Movasaghi et al., 2008). Whereas, during PF-SeNPs biosynthesis course, broad intense peak at 3,382 cm^-1^ of aqueous fruit extract of *E. officinalis* was shifted to 3,348 cm^-1^ of PF-SeNPs, which suggested that selenium has interacted with the hydroxyl group from aqueous fruit extract of *E. officinalis* through hydrogen bonding and facilitated biosynthesis of PF-SeNPs. Likewise, prominent peak at 1,718 cm^-1^ (carbonyl C=O stretch) of aqueous fruit extract of *E. officinalis* has disappeared in PF-SeNPs, which specify that carbonyl C=O stretch has enabled the synthesis of PF-SeNPs (Ankamwar et al., 2005). Similarly, peak at 1,651 cm^-1^ (amide I vibrations) in aqueous fruit extract of *E. officinalis* was shifted to higher frequencies 1,662 cm^-1^ in PF-SeNPs biosynthesis, which shows the interaction of proteins of aqueous fruit extract of *E. officinalis* with selenium through the amine groups. The large and intense peak at 1,050 cm^-1^ in aqueous fruit extract of *E. officinalis* was shifted to 1,042 cm^-1^ in PF-SeNPs, which represents the characteristic Se–O stretching vibration, according to the report of Kannan et al. (2014) and accomplishes the successful biosynthesis of PF-SeNPs. According to Kimura et al. (2005), peaks at 1,050 cm^-1^ (C-O), 1,540 cm^-1^ (C-N-H) and 1,660 cm^-1^ (HN-H) are indicative of carbohydrate and protein character, respectively. FTIR analysis indicated that proteins and carbohydrates were dominant on the surface of PF-SeNPs. The aforementioned variations at the peaks show that secondary metabolites of aqueous fruit extract of *E. officinalis* have successfully facilitated the biosynthesis of PF-SeNPs by reduction process and could aid in protection of PF-SeNPs from aggregation and thereby retain their long-term stability (Park et al., 2011).

**Figure 4:**
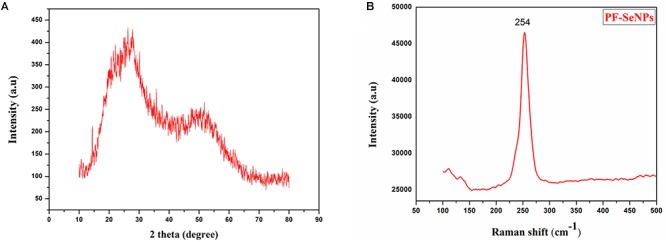
**(A)** X-ray diffraction (XRD) pattern and **(B)** Raman spectroscopy of phytofabricated selenium nanoparticles (PF-SeNPs).

The nature of the synthesized PF-SeNPs was assessed by XRD and Raman spectroscopic analysis. The XRD pattern of PF-SeNPs has been depicted in Figure 4A, which shows broader peak without any sharp Bragg’s peaks and thus, it specifies that synthesized red elemental PF-SeNPs is certainly amorphous in nature, which is in agreement with previous reports. Li et al. (2007) and Yang et al. (2012) have phyto-synthesized SeNPs correspondingly from *Capsicum annuum* leaf extract and *Spirulina* polysaccharide and reported its nature as amorphous. The attained XRD results were further evaluated by Raman spectroscopic analysis. Raman spectroscopy provides the unique feature of detecting vibrational characteristics of the synthesized nanomaterials, which are amorphous or crystalline in nature and it also has been proven to be a powerful tool to analyze the phase of nanoparticles (Baganich et al., 1991). In the present study, characteristic resonance peak was observed at 254 cm^-1^ (Figure 4B), and it could be attributed to amorphous selenium as a consequence of irregularly and arrayed selenium atoms as disordered chains (Li et al., 2010).

**Figure 5:**
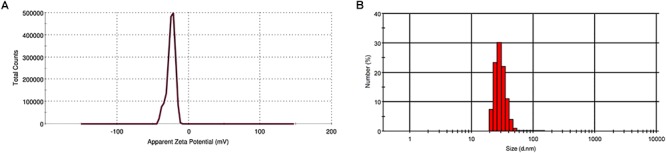
**(A)** Zeta potential (mV) and **(B)** size distribution (d.nm) of phytofabricated selenium nanoparticles (PF-SeNPs).

The stability and hydrodynamic size of PF-SeNPs were assessed by Zeta potential and DLS analysis. Zeta potential is the measure of an effective electric charge on the surface of nanoparticles. The magnitude of the Zeta potential delivers information on particle stability. The nanoparticles with higher magnitude of Zeta potential exhibits increased stability due to greater electrostatic repulsion between nanoparticles. In the present study, the sharp peak of Zeta potential for PF-SeNPs was noticed at -24.4 mV, and it was concluded that surface of PF-SeNPs is negatively charged (Figure 5A). The negative charged potential value was imparted to PF-SeNPs because of reducing agents (phenolics, flavonoids, and tannins) of aqueous fruit extract of *E. officinalis*. Also, the attendance of negative electro-static forces between PF-SeNPs favors to exist in dispersion form (Kokila et al., 2017). Further, the DLS pattern reveals that the optimized SeNPs synthesized by this method have a size ranging from 20 to 60 nm and display a narrow peak (Figure 5B), which indicates nanoparticles are monodispersed and having polydispersity index less than 0.2, which shows the lesser aggregation of particles (Bhattacharjee, 2016).

**Figure 6:**
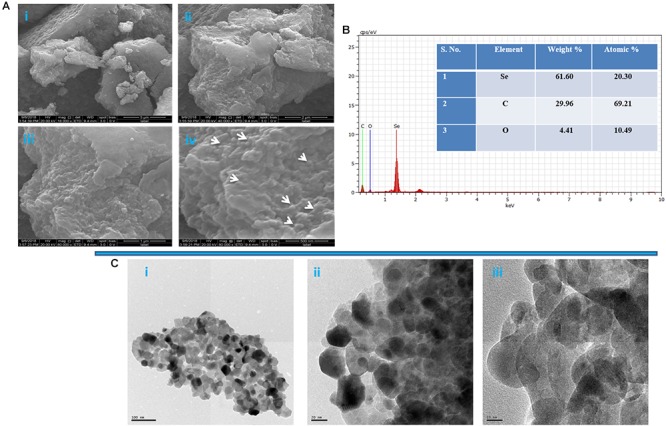
**(A)** Scanning electron microscopic image of phytofabricated selenium nanoparticles (PF-SeNPs) at magnification of (i) 5 μm, (ii) 2 μm, (iii) 1 μm, and (iv) 500 nm. **(B)** Energy-dispersive X-ray analysis of PF-SeNPs. **(C)** Transmission electron microscopic images of PF-SeNPs at (i) 100 nm, (ii) 20 nm, and (iii) 10 nm.

The morphology of SeNPs was confirmed by SEM analysis and was found to be spherical (Figure 6A). The existence of three strong peaks at 1.5 keV of EDX spectrum confirm the presence of elemental Se (61.60%), C (29.96%) and O (4.41%) (Figure 6B). The shape and size of PF-SeNPs were confirmed by HR-TEM analysis, and it was found to be spherical shape with an average diameter of 15–40 nm (Figure 6C). The obtained HR-TEM results were in accordance with DLS and SEM assessments of PF-SeNPs. However, the sizes of the PF-SeNPs were slightly higher in DLS related to HR-TEM analysis, and it could be due to hydrodynamic coating of water molecules around PF-SeNPs (Eaton et al., 2017). In support of our study, Li et al. (2007), Yang et al. (2012), Ramamurthy et al. (2013), Kong et al. (2014), Sharma et al. (2014) and have bio-synthesized SeNPs from *Capsicum annuum* L. extract, *Spirulina* polysaccharide, fenugreek seed extract, Arabic gum, *Vitis vinifera* dried fruit extract, and observed optimum size of SeNPs as 80, 90–550, 50–150, 34 and 3–18 nm, respectively. The size of bio-synthesized nanoparticles relies on chemical constituents of the biomaterial employed (Akhtar et al., 2013). Therefore, the size of PF-SeNPs in our study was quite diverse related to earlier reports.

### Bio-Potentials of PF-SeNPs

#### Antioxidant Activity

**Figure 7:**
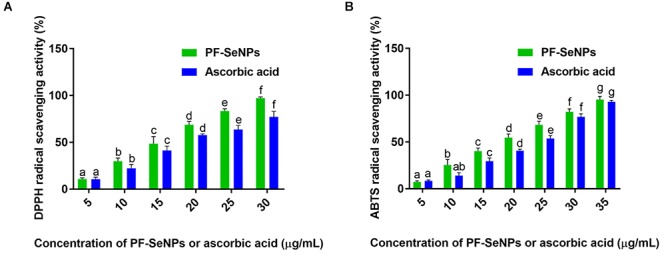
Determination of dose-dependent antioxidant potential of phytofabricated selenium nanoparticles (PF-SeNPs) and ascorbic acid by **(A)** DPPH and **(B)** ABTS radical scavenging assays. The experiment was executed independently in triplicates and data was expressed as mean ± SD. The statistical difference between the test samples were assessed by Tukey’s multiple comparison test and *p* ≤ 0.05 was considered as a significant. The bar graphs with different alphabetic letters within the respective study were significant.

Antioxidant activity of PF-SeNPs was assessed by DPPH and ABTS free radical scavenging assays. The PF-SeNPs has shown potent DPPH and ABTS radical scavenging activity and it was exhibited in dose-dependent manner (Figure 7A,B). It shows that antioxidant activity was directly proportional to the concentration of PF-SeNPs. The EC50 (effective concentration required to inhibit 50% of free radicals) of PF-SeNPs was determined as 15.67 ± 1.41 and 18.84 ± 1.02 μg/mL for DPPH and ABTS radical scavenging activity, respectively. Whereas, EC50 value of ascorbic acid (reference standard) was noticed as 19.21 ± 2.63 and 21.69 ± 1.77 μg/mL for DPPH and ABTS radical scavenging activity, respectively. The PF-SeNPs has exhibited potent antioxidant activity compared to ascorbic acid. Our results were in line and comparable with the reports of phyto-synthesized SeNPs. Recently, Kokila et al. (2017) have phyto-synthesized SeNPs of 16 nm size and noticed their EC50 value as 22.5 μg/mL. In another report, Qiu et al. (2018) have synthesized pectin decorated SeNPs of 41 nm and quantified its EC50 value as 500 μg/mL. The aforementioned studies strongly support that the antioxidant activity of PF-SeNPs is dependent not only on size and but also on surface functional moieties of nanoparticles that are occupied by secondary metabolites of *E. officinalis* fruit extract (Zhang et al., 2004; Singh et al., 2018).

The higher antioxidant property of PF-SeNPs could be due to selenium as well, which plays a key role in an upturn of selenoenzymes like glutathione peroxidase and assist in protecting the cells and tissue under *in vivo* conditions from free radicals (Rotruck et al., 1973). Consequently, the physicians and nutritionists routinely recommend to consume Se-rich food sources (nuts, cereals and mushrooms) for keeping the individual fit (Combs and Combs, 1986). Therefore, preparation of nanoparticles with Se and augmentation of their bio-functions by phytofabrication is much anticipated and accepted by the consumer, government, and regulatory agencies (El-Ramady et al., 2014). Therefore, bio-synthesized PF-SeNPs could be considered as highly biocompatible, and it could find a potential role in substituting the synthetic antioxidants and could be used as an natural antioxidant embedding agent in food packaging material (Vera et al., 2016; Wadhwani et al., 2016).

#### Antimicrobial Activity

Selenium is considered as potent antimicrobial agents, and its derivative substance like selenium sulfide is widely used in medicine to treat infections of *Malassezia* and *Tinea versicolor* (Pierard et al., 1997). However, excess use of selenium causes toxic effects and leads to selenosis (Spallholz, 1994). Consequently, contemporary research has focused on diminishing the toxicity and improving the bio-functional aspects of selenium. In turn, nanotechnology has provided the safe strategy to reduce the toxicity and improve the bio-functionality of selenium through biosynthesis.

In the present study, the antimicrobial activity of biosynthesized PF-SeNPs was tested against foodborne pathogens by micro-well dilution technique as per instructions of CLSI. To the best of our knowledge, previous studies were mostly focused on an evaluation of the MIC activity of bio-synthesized PF-SeNPs. Only a few studies were focused on the evaluation of MBC and MFC value of biosynthesized PF-SeNPs. In medical microbiology, determination of MBCs and MFCs has been encouraged for the treatment of serious microbial infections (i.e., endocarditis) or treatment of immunosuppressed patients (Sykes, 2013).

The biogenic PF-SeNPs have presented potent antimicrobial activity on both bacterial and fungal pathogens (Table 1). In the case of antibacterial activity, MIC and MBC values of PF-SeNPs were in the range of 09.16 ± 0.76–59.83 ± 2.56 and 19.83 ± 1.25–97.50 ± 3.27 μg/mL, respectively. The lowest MIC and MBC values of PF-SeNPs were correspondingly noticed as 09.16 ± 0.76 and 19.83 ± 1.25 μg/mL against *S. aureus* MTCC 96. While, highest MIC and MBC values of PF-SeNPs were correspondingly noticed as 59.83 ± 2.56 and 97.50 ± 3.27 μg/mL against *E. coli* MTCC 41. Together, the potent antibacterial activity of PF-SeNPs was observed against Gram-positive bacteria (*S. aureus* MTCC 96, *E. faecalis* MTCC 439 and *L. monocytogenes* MTCC 657) compared to Gram-negative bacteria (*E. coli* MTCC 4). On the other hand, PF-SeNPs exhibited potent antifungal activity compared to antibacterial activity. The MIC and MFC values of PF-SeNPs on fungi were observed in the range of 07.50 ± 1.32–25.50 ± 2.78 and 10.67 ± 1.04–38.17 ± 1.75 μg/mL, respectively. The least MIC and MFC of PF-SeNPs were correspondingly observed as 07.50 ± 1.32 and 10.67 ± 1.04 μg/mL against *R. stolonifer* MTCC 4886. The highest MIC and MFC of PF-SeNPs were correspondingly observed as 25.50 ± 2.78 and 38.17 ± 1.75 μg/mL against *A. oryzae* MTCC 634.

**Table 1:** Antimicrobial effect of phytofabricated selenium nanoparticles (PF-SeNPs) from *E. officinalis* fruit extract on various foodborne pathogens determined by micro-well dilution technique.

Microbial culture	MIC^@^ (μg/mL)	MBC^#^/MFC^$^ (μg/mL)
		
Bacteria		
*Escherichia coli* MTCC 41	59.83 ± 2.56^a^	97.50 ± 3.27^a^
*Listeria monocytogenes* MTCC 657	33.17 ± 2.84^b^	53.50 ± 3.04^b^
*Staphylococcus aureus* MTCC 96	09.16 ± 0.76^c^	19.83 ± 1.25^c^
*Enterococcus faecalis* MTCC 439	16.17 ± 2.02^d^	33.17 ± 3.32^d^
		
Fungi		
*Aspergillus brasiliensis* MTCC 1344	07.66 ± 1.52^a^	11.83 ± 0.76^a^
*A. flavus* MTCC 1883	13.50 ± 1.32^b^	20.50 ± 2.29^b^
*A. oryzae* MTCC 634	25.50 ± 2.78^c^	38.17 ± 1.75^c^
*A. ochraceus* MTCC 10276	13.33 ± 1.89^bd^	21.00 ± 1.32^bd^
*Fusarium anthophilum* MTCC 10129	11.83 ± 0.28^abde^	22.17 ± 1.60^bde^
*Rhizopus stolonifer* MTCC 4886	07.50 ± 1.32^aef^	10.67 ± 1.04^af^

^@^*MIC, minimum inhibitory concentration; ^#^MBC, minimum bactericidal concentration; ^$^MFC, minimum fungicidal concentration. The statistical difference between the test samples within MIC and MBC/MFC of the bacteria and fungi were assessed by Tukey’s multiple comparison test. The *p* ≤ 0.05 was considered as significant. The different alphabetic letters within MIC and MBC/MFC of the bacteria and fungi were significant*.

In support of our study, reports are scarce on antimicrobial activity of biosynthesized SeNPs using plant extracts. Kokila et al. (2017) have biosynthesized SeNPs using leaf extract and reported their antimicrobial activity in the form of zone of inhibition as 08, 07, and 08 mm against *S. aureus*, *E. coli*, and *A. niger*, respectively. Alternatively, few reports are available on the exploration of biosynthesized SeNPs using microbial sources. Zonaro et al. (2015) have biosynthesized SeNPs from *Stenotrophomonas maltophilia* and noticed MIC values of 125 mg/L against *E. coli* JM109 and *E. coli* ATCC 25922, and 250 mg/L against *P. aeruginosa* PAO1, *P. aeruginosa* ATCC 27853 and *S. aureus* ATCC 25923. In case of antifungal activity, Shahverdi et al. (2010) have synthesized SeNPs from *Klebsiella pneumonia* and observed complete inhibition of visible fungal growth in the range of 10–260 μg/mL against *M. sympodialis, M. furfur*, and *A. terreus*. In the same way, Kazempour et al. (2013) have biosynthesized SeNPs from *Klebsiella pneumonia* and estimated their MIC values as 250 and 2,000 μg/mL toward *A. brasiliensis* and *Candida albicans*, respectively.

In earlier reports, researchers have established that biosynthesized nanoparticles elicit antimicrobial effects through different ways. The antimicrobial activity depends on the size of nanoparticles. The small size of nanoparticles can effortlessly cross the cell wall and membrane and induce cell lysis. It further, interferes with respiratory sequence and ATP synthesis, and stops cell division and induce the microbial cell death (Zonaro et al., 2015).

Our study has shown potent antimicrobial activity against Gram-positive bacteria compared to Gram-negative bacteria. To explain this, Tran et al. (2015) have assessed the antibacterial mechanism of SeNPs in Gram-positive and Gram-negative bacteria and concluded that SeNPs have strong electrostatic repulsion toward the lipopolysaccharide and membrane of Gram-negative bacteria, which is highly negative in nature. Whereas, Gram-positive bacteria have considerably less negative charge than Gram-negative bacteria. So, there is a possibility of higher deposition of SeNPs on the surface of Gram-positive bacteria to induce bacterial death. Therefore, Gram-negative bacteria tend to show resistance to PF-SeNPs. Interestingly, PF-SeNPs have presented potent antimicrobial activity against fungi rather than bacteria. The selenium is considered as the strong antifungal agent rather than antibacterial, and hence, it is highly used in anti-dandruff shampoo to treat fungal infections (Pierard et al., 1997). Therefore, in our study, strong antifungal activities of PF-SeNPs were noticed. Until date, the exact mechanism involved in an antifungal activity of selenium is unknown.

#### Biocompatibility Assay

Biocompatibility of synthesized PF-SeNPs was appraised by comparing with the cytotoxicity aspects of sodium selenite in N2a cells, and it was done by cell viability, MMP and caspase-3 assays.

**Figure 8:**
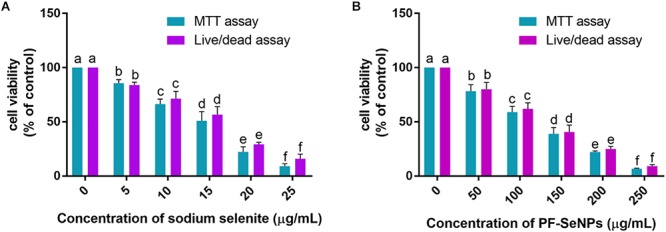
Determination of dose-dependent inhibitory effect of **(A)** sodium selenite and **(B)** phytofabricated selenium nanoparticles (PF-SeNPs) on cell viability of N2a cells for 24 h by MTT and live/dead dual staining assays. The experiments were executed independently in triplicates and data was expressed as mean ± SD. The statistical difference between the test samples were assessed by Tukey’s multiple comparison test and *p* ≤ 0.05 was considered as a significant. The bar graphs with different alphabetic letters within the respective study were significant.

**Figure 9:**
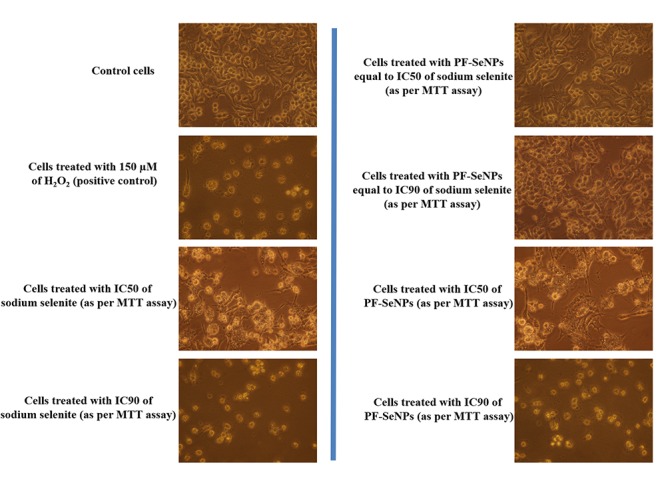
Bright-field microscopic images of N2a cells treated with 150 μM of H_2_O_2_ and different concentrations of sodium selenite and phytofabricated selenium nanoparticles (PF-SeNPs) for 24 h. All images were captured at magnification of 400×.

**Figure 10:**
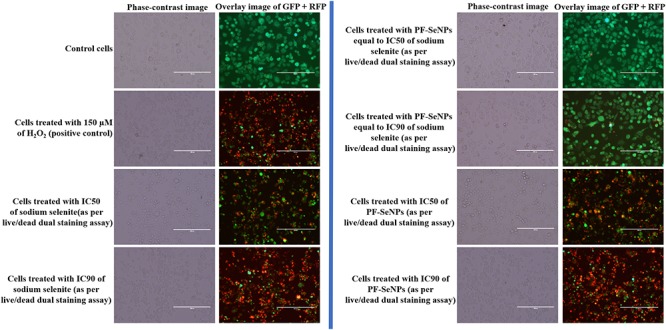
Live/dead dual staining images of N2a cells treated with 150 μM of H_2_O_2_ and different concentrations of sodium selenite and phytofabricated selenium nanoparticles (PF-SeNPs) for 24 h. The scale bar is 200 μm.

The cell viability was considered by MTT and live/dead dual staining assays. The MTT assay measure the cell viability as a function of redox potential of cell and metabolically active cells convert MTT reagent to purple formazan crystals and its strength is directly proportional to viable cells (Kalagatur et al., 2018b). The other assay, live/dead dual staining is an advanced cell viability technique and it consists of two staining reagents, i.e., calcein AM and ethidium homodimer-1. The calcein AM is used to stain live cells and it actively passes through cellular membrane and emits green fluorescence at an excitation of 495 nm and emission of 515 nm by action of ubiquitous intracellular esterases of live cells. The other dye, ethidium homodimer-1 is specific for dead cells and it cross only through compromised cellular membrane and strongly binds to nuclear material of cell and emits red fluorescence at an excitation of 495 nm and emission of 635 nm (Kalagatur et al., 2018b). In the present study, MTT and live/dead dual staining assays concluded that sodium selenite and PF-SeNPs have inhibited the cell viability in dose-dependent way (Figure 8). However, sodium selenite has inhibited cell viability at much lower concentration compared to PF-SeNPs. The IC50 (dose required to inhibit 50% of cell viability) and IC90 (dose required to inhibit 90% of cell viability) values were calculated to assess the cytotoxicity of sodium selenite and PF-SeNPs. In MTT assay, IC50 and IC90 values for sodium selenite and PF-SeNPs were observed as 14.01 ± 1.88 and 24.60 ± 2.19 μg/mL, 127.28 ± 3.73 and 234.41 ± 5.57 μg/mL, respectively. In live/dead dual staining assay, IC50 and IC90 values for sodium selenite and PF-SeNPs were noticed as 15.30 ± 2.08 and 27.11 ± 1.92 μg/mL, 132.66 ± 4.02 and 241.83 ± 4.79 μg/mL, respectively. Meanwhile, 150 μM of H_2_O_2_ inhibited 92.38 ± 2.91% of cell viability as determined by MTT assay (graph not shown) and was considered as positive control. The observed inhibitory concentrations of sodium selenite and PF-SeNPs were much like in both MTT and live/dead dual staining assays. The IC50 and IC90 values of PF-SeNPs was much higher compared to sodium selenite and concluded that PF-SeNPs was less toxic than sodium selenite. To assess further, bright-field and live/dead fluorescent microscopic images were depicted in Figure 9, 10, respectively. The bright-field images have clearly showed cellular membrane rupture and apoptotic bodies in cells treated with IC50 values of sodium selenite and PF-SeNPs. Relatively, massive cellular membrane damage and high apoptotic bodies formation were noticed at IC90 values of sodium selenite and PF-SeNPs (Figure 9). Similarly, live/dead dual staining assay has also determined comparable pattern of results with bright-field microscopic images. At IC50 dose of sodium selenite and PF-SeNPs, about half live (green) and dead (red) cells were noticed and as usually quite higher number of dead (red) cells was noticed at IC90 concentrations of sodium selenite and PF-SeNPs (Figure 10). Interestingly, cells treated with PF-SeNPs equal to IC50 and IC90 doses of sodium selenite has shown negligible changes in bright-field and live/dead fluorescent images compared to control cells (Figure 9, 10). Thus, cell viability assays concluded that PF-SeNPs was less toxic and much safer compared to sodium selenite. Our results were well supported by earlier reports, Anu et al. (2017) has compared the cytotoxic effects of chemically synthesized and green-synthesized of SeNPs using *Allium sativum* on Vero cells and reported that green-synthesized SeNPs were less toxic than chemically synthesized SeNPs. Similarly, Forootanfar et al. (2014) have biosynthesized SeNPs from marine bacterial isolate *Bacillus* sp. MSh-1 and noticed lower cytotoxic effect in MCF-7 cells related to selenium dioxide.

**Figure 11:**
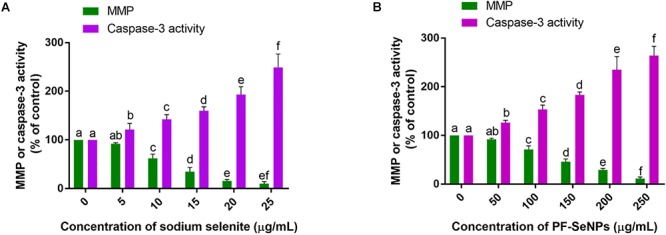
Effect of different concentration of **(A)** sodium selenite and **(B)** phytofabricated selenium nanoparticles (PF-SeNPs) on mitochondrial membrane potential (MMP) and caspase-3 activity of N2a cells for 24 h. The experiments were executed independently in triplicates and data was expressed as mean ± SD. The statistical difference between the test samples were assessed by Tukey’s multiple comparison test and *p* ≤ 0.05 was considered as a significant. The bar graphs with different alphabetic letters within the respective study were significant.

**Figure 12:**
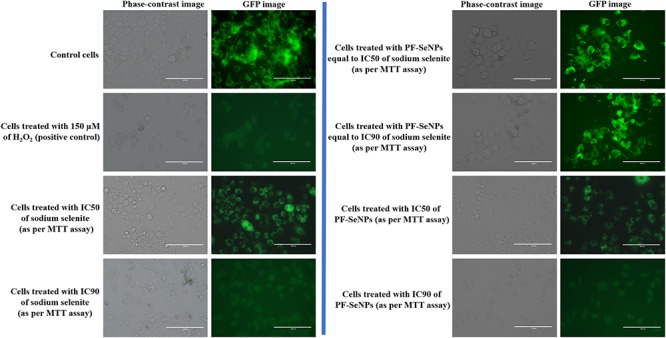
Rhodamine 123 stained images depicting mitochondrial membrane potential (MMP) of N2a cells treated with 150 μM of H_2_O_2_ and different dose of sodium selenite and phytofabricated selenium nanoparticles (PF-SeNPs) for 24 h. The scale bar is 100 μm.

The molecular mechanisms involved in the cytotoxicity was revealed by MMP and caspase-3 assays. Upholding the MMP is crucial for ATP synthesis and other cellular functions, and its depletion induces the cell death. The level of MMP is routinely assessed by rhodamine 123 staining and its fluorescence strength is considered as directly proportional to the level of MMP (Kalagatur et al., 2017). On the other hand, caspase-3 is a caspase protein and its activation induce the cell death by apoptosis process (Porter and Jänicke, 1999). In the present study, sodium selenite and PF-SeNPs have depleted the MMP and elevated the caspase-3 activity in dose-dependent manner (Figure 11). The study concluded that sodium selenite and PF-SeNPs induce the cytotoxicity by apoptosis process through depleting MMP levels. However, sodium selenite has depleted the MMP and elevated the caspase-3 activity at much lower levels compared to PF-SeNPs and these results were in accordance the outcome of cell viability assays. Most importantly, cells treated with PF-SeNPs of IC50 and IC90 values of sodium selenite have not shown much reduction in fluorescence (MMP) compared to control (Figure 12). The obtained cytotoxicity of PF-SeNPs was found to be comparable with earlier reports (Table 2). Thus, biocompatibility study concluded that biosynthesized PF-SeNPs were much less toxic and safer in comparison to sodium selenite.

**Table 2:** Overview of *in vitro* cytotoxicity of selenium nanoparticles (SeNPs) on different cell lines.

S. No.	Precursor	Reducing agent	Stabilizing agent	Cell line	IC50 value	References
1	Sodium selenite	*Psidium guajava* leaf extract	–	CHO pro (hamster ovary)	88 ± 2.1 μg/mL	Alam et al., 2018
2	Sodium selenite	Ascorbic acid	Dextrin	NIH-3T3 (mouse embryonic fibroblast)	149 ± 60.5 μg/mL	Malhotra et al., 2016
3	Sodium selenite	Ascorbic acid	*Ganoderma lucidum* polysaccharides	Raw 264.7 (mouse macrophage)	5–100 μg/mL	Wang et al., 2014
4	Sodium selenite	*Halococcus salifodinae* BK18	–	HaCat (human skin)	150 μg/mL	Srivastava et al., 2014
5	Sodium selenite	*Gracilaria lemaneiformis* polysaccharides	–	Chem-5 (human brain glial)	159.9 ± 9.73 μM	Jiang et al., 2014
1.				HK-2 (human kidney)	79.5 ± 4.26 μM	
0.				L02 (human liver)	95.6 ± 7.68 μM	
6	Selenium dioxide	*Bacillus* sp. MSh-1	–	MCF-7 (human mammary gland)	41.5 ± 0.9 μg/mL	Forootanfar et al., 2014
7	Selenous acid	Ascorbic acid	Chitosan	HK-2 (human kidney)	98.29 μM	Yu et al., 2012
8	Selenium dioxide	Ascorbic acid	Sialic acid	HK-2 (human kidney)	40 μg/mL	Zheng et al., 2011
9	Sodium selenite	Ascorbic acid	*Undaria pinnatifida* polysaccharides	Hs68 (human skin fibroblast)	67.9 μM	Chen et al., 2008

## Conclusion

The present study shows phytofabrication of SeNPs from aqueous fruit extract of *E. officinalis* by facile, green, economic, and eco-friendly approach. The aqueous fruit extract of *E. officinalis* was rich with phenolics, flavonoids, and tannins, and was found appropriate for biosynthesis of nanoparticles. The synthesized PF-SeNPs exhibited highly stable, negative charge, amorphous nature, spherical shape and nano-size. The PF-SeNPs has presented efficient bio-potential applications, i.e., antioxidant, antimicrobial, and biocompatibility. The PF-SeNPs has shown potent free radical scavenging activity and it was found highly efficient than standard antioxidant ascorbic acid. Also, PF-SeNPs shown potent antimicrobial activity on wide range of foodborne pathogens and it was found highly efficient on fungi followed by Gram-positive and Gram-negative bacteria. The PF-SeNPs have exhibited lesser toxicity on N2a cells compared to sodium selenite, rendering them highly safer and biocompatible. Therefore, with these bio-potential impacts, the PF-SeNPs have a tremendous application to be applied in the pharmaceutical, biomedical and food industries, and exclusively as an antimicrobial and antioxidant agent.

## Author Contributions

LG and RD have designed the work. LG, RD, and NK executed the experiments, drafted the results, and approved the final version of the manuscript.

## Conflict of Interest Statement

The authors declare that the research was conducted in the absence of any commercial or financial relationships that could be construed as a potential conflict of interest.
